# Verification of the Accuracy of Cervical Spinal Cord Injury Prognosis Prediction Using Clinical Data-Based Artificial Neural Networks

**DOI:** 10.3390/jcm13010253

**Published:** 2024-01-01

**Authors:** Jun Kishikawa, Kazu Kobayakawa, Hirokazu Saiwai, Kazuya Yokota, Kensuke Kubota, Tetsuo Hayashi, Yuichiro Morishita, Muneaki Masuda, Hiroaki Sakai, Osamu Kawano, Yasuharu Nakashima, Takeshi Maeda

**Affiliations:** 1 Department of Orthopedic Surgery, Spinal Injuries Center, Fukuoka 820-8508, Japan; jun0627k@gmail.com (J.K.); yokota@ortho.med.kyushu-u.ac.jp (K.Y.); kkubota8@ortho.med.kyushu-u.ac.jp (K.K.); tetsuo884hayashi@yahoo.co.jp (T.H.); uchiro1968@mac.com (Y.M.); sudarax@mac.com (M.M.); hiro_yale@yahoo.co.jp (H.S.); orthosic@gmail.com (O.K.); maeken@gd6.so-net.ne.jp (T.M.); 2 Department of Orthopedic Surgery, Kyushu University, Fukuoka 812-8582, Japan; saiwai.hirokazu.255@m.kyushu-u.ac.jp (H.S.); nakashima.yasuharu.453@m.kyushu-u.ac.jp (Y.N.)

**Keywords:** artificial neural networks (ANNs), cervical spinal cord injury, prognosis prediction, rehabilitation, artificial intelligence

## Abstract

Background: In patients with cervical spinal cord injury (SCI), we need to make accurate prognostic predictions in the acute phase for more effective rehabilitation. We hypothesized that a multivariate prognosis would be useful for patients with cervical SCI. Methods: We made two predictive models using Multiple Linear Regression (MLR) and Artificial Neural Networks (ANNs). We adopted MLR as a conventional predictive model. Both models were created using the same 20 clinical parameters of the acute phase data at the time of admission. The prediction results were classified by the ASIA Impairment Scale. The training data consisted of 60 cases, and prognosis prediction was performed for 20 future cases (test cohort). All patients were treated in the Spinal Injuries Center (SIC) in Fukuoka, Japan. Results: A total of 16 out of 20 cases were predictable. The correct answer rate of MLR was 31.3%, while the rate of ANNs was 75.0% (number of correct answers: 12). Conclusion: We were able to predict the prognosis of patients with cervical SCI from acute clinical data using ANNs. Performing effective rehabilitation based on this prediction will improve the patient’s quality of life after discharge. Although there is room for improvement, ANNs are useful as a prognostic tool for patients with cervical SCI.

## 1. Introduction

Patients with cervical spinal cord injury (SCI) experience varying degrees of paralysis. Generally, patients who develop mild paralysis in the early stages have a good recovery. However, even if severe paralysis develops early, there may be significant recovery, making it difficult to predict the prognosis [1]. This difficulty in predicting makes it difficult to set rehabilitation goals for patients. Therefore, accurately predicting each patient’s motor function prognosis at the early stages of injury is important for rehabilitation goals and post-discharge life planning [2]. If we can predict the prognosis early after injury, it will be possible to perform rehabilitation according to the functional outcome. For example, if there is a high possibility that the patient will be able to walk, rehabilitation with a walker will be necessary, and if severe paralysis remains and it is difficult to stand up, rehabilitation using a wheelchair will be necessary. In addition, the number of patients with cervical SCI is increasing in Japan year by year; therefore, it is expected that it will be more important to perform efficient rehabilitation and that patients will return to society or be discharged from the hospital as soon as possible [3]. For this reason, it is very important to accurately predict the prognosis of cervical SCI from the early stages of injury.

Furthermore, accurate prognosis prediction plays an important role in determining the effectiveness of new treatments. Currently, a lot of basic research is being carried out on SCI patients, and some treatments are already at the stage of clinical application [4,5]. Unlike basic research, it is extremely difficult to establish an accurate control group in clinical research. If accurate prognosis prediction becomes possible, we can use the prediction results as a control to determine the effectiveness of new treatments, which is a huge advantage for the future development of SCI research. However, it is difficult to accurately predict the prognosis at an early stage of injury [6]

So far, many studies have been conducted on predicting the prognosis of patients with cervical SCI, and many univariate factors have been reported (e.g., blood glucose [7], Zinc [8], OPLL [9,10]). However, these factors are intricately intertwined and influence the final prognosis. In addition, patients come from a wide range of backgrounds, from young to elderly, and have a wide range of complications, and the types of trauma and associated fractures also affect prognosis. Therefore, prognosis prediction using univariate variables is extremely difficult, and prediction using multivariate variables is highly anticipated. So, we made a hypothesis that combining these univariate factors and predicting prognosis using multivariate factors would enable more accurate prognosis prediction. In fact, an accurate prognosis prediction method using a multivariate analysis has not yet been established. Conventionally known multivariate prediction models include those based on Multiple Linear Regression (MLR) analysis, but the prediction accuracy of this model remains limited [11,12]. Therefore, we wondered if it was possible to create a predictive model that was better than this conventional MLR analysis model.

**Figure 1:**
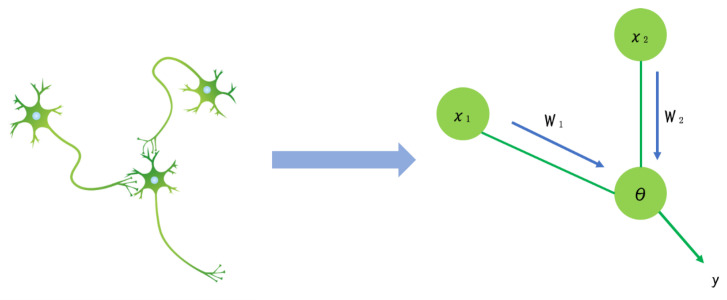
ANNs are inspired by the sophisticated functionality of human brains.

**Figure 2:**
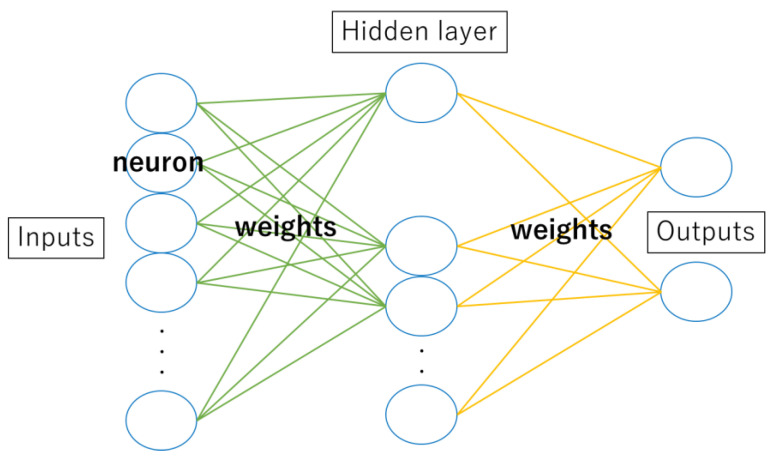
They are comprised of a large number of connected nodes, each of which performs a simple mathematical operation.

In the various multivariate models, Artificial Neural Networks (ANNs) are one of the AI machine learning models. ANNs are inspired by the sophisticated functionality of human brains, where hundreds of billions of interconnected neurons process information in parallel (Figure 1) [13]. They are comprised of a large number of connected nodes, each of which performs a simple mathematical operation (Figure 2). ANNs are currently being applied in many fields, including medical science [14,15]. For instance, for breast, colorectal, prostate, and hypopharyngeal cancer, accurate prognostic predictions have been made by using ANNs, and its usefulness in the future is highly expected [16,17,18]. Therefore, we used ANNs to construct a model that enables an accurate prognosis of cervical SCI in the acute phase.

Currently, prognosis predictions for patients with cervical SCI are often made by individual physicians based on experience. We hope that this research will enable many people to predict the prognosis of patients with SCI more easily and help determine subsequent treatment strategies. We created two predictive models using MLR analysis and ANNs from the same 20 parameters. We compared them to determine which model was better.

## 2. Materials and Methods

### 2.1. Participants and Data Splitting

Eighty-six individuals were transported to Spinal Injuries Center (SIC in Fukuoka, Japan) by the day after the injury from February 2013 to May 2019. In 6 cases of 86, neurological evaluation was insufficient, so these 6 cases were excluded. Therefore, the cohort consisted of 80 patients. We defined the early 60 cases as training cohorts and later 20 cases as test cohorts (Table 1). By dividing the cases chronologically, we aimed for a pseudo-prospective study. The average age of the training cohort was 61.9 years (range: 18–83), with 47 (78.3%) men and 13 (21.7%) women. Meanwhile, the average age of the test cohort was 56.1 years (range: 18–79), with 17 cases (85.0%) being male and 3 cases (15.0%) being female. The number of cases by ASIA Impairment Scale upon admission in the training cohort was 31 cases (38.8%) of grade A, 15 cases (18.8%) of grade B, 30 cases (37.5%) of grade C, and 4 cases of grade D (5.0%).

In order to build machine learning models, learning using training datasets is essential. Training data are also called learning data. A test cohort is a data set used to check the accuracy of the constructed machine learning model and uses data that are not used in the training set to perform accuracy tests. The training cohort was transported to SIC from July 2017 to May 2019, and the test cohort was delivered after June 2019. All patients were hospitalized for 6 months or more. In the hospital, they underwent SIC rehabilitation program.

### 2.2. Primary Endpoint

In this study, the primary endpoint was the ASIA Impairment Scale (AIS) at hospital discharge. All patients were discharged more than 6 months after the injury. AIS is a medical rating scale for assessing the degree of SCI. This scale is useful in classifying the degree of symptoms and functional impairment in patients with SCI and in developing treatment and rehabilitation plans. It also helps to track the progression of symptoms and monitor the effectiveness of treatment.

### 2.3. Variables: Clinical Parameters

We retrospectively investigated clinical parameters, including age at injury, sex, height, body weight, lesion type, ossification of the posterior longitudinal ligament (+/−), sagittal low in high (+/−), pre-vertebral hematoma (+/−), *AIS (ASIA Impairment Scale on admission), **NLI (Neurological Level of Injury), ***ZPP (Zone of Partial Preservation), ****ASIA total motor score on admission, blood pressure, diabetes (+/−), white blood cell count, neutrophil count, monocytes count, platelet count, C-reactive protein (CRP), and blood glucose.

*AIS (ASIA Impairment Scale) is a tool developed by the American Spinal Injury Association (ASIA) to assess neurological outcomes in patients with spinal cord injuries. AIS is classified into five levels from grade A to E. Grade A = Complete. Grade B = Sensory Incomplete. Grade C and D = Motor Incomplete. Grade E = Normal.

**NLI (Neurological Level of Injury): “The most caudal segment of the spinal cord with normal sensory and antigravity motor function on both side of the body”.

***ZPP (Zone of Partial Preservation): “The most caudal segment with some sensory and motor levels with some sensory and/or motor function defines the extent of the sensory or motor ZPP respectively and are documented as four distinct levels (R-sensory, L-sensory, R-motor, and L-motor)”.

****ASIA total motor score: “Normal strength is assigned a grade of 5 for each muscle function. A score of 5 for each of the five key muscle functions of the upper extremity would result in a maximum score of 25 for each extremity, totaling 50 for the upper limbs. The same is true for the five key muscle functions of the lower extremity, totaling a maximum score of 50 for the lower limbs”. From the above, the total motor score is 100 for the upper and lower limbs.

These definitions are taken from the International Standards for Neurological Classification of Spinal Cord Injury (ISNCSCI) (Revised 2019) [19,20].

### 2.4. Methods

#### 2.4.1. Multiple Linear Regression (MLR)

As a conventional predictive model using multivariate analysis, we used MLR analysis to create a prediction profile. We adopted Multiple Linear Regression in JMP^®^ Pro version 14.2.0 (SAS Institute Inc., Cary, NC, USA). This analysis was conducted using the variables described above. We created a prediction profile using the 60 cases as a training cohort. By inputting various parameters into the profile, it is possible to predict the final prognosis at the time of discharge in the test cohort.

#### 2.4.2. Artificial Neural Networks (ANNs)

ANNs, one of the supervised machine learnings inspired by the biological neural networks in human brain, were used in this study. We adopted neural networks in JMP^®^ Pro version 14.2.0 (SAS Institute Inc., Cary, NC, USA), and the variables used were 20 parameters similar to those used in MLR analysis. Weight Decay is used as a penalty method to reduce the risk of overfitting by limiting the degrees of freedom of a parameter while preserving the predictive model’s ability. It consists of input, 1st, 2nd, and output layers. The number of nodes in the 1st and 2nd layers are 5 and 10, respectively. First, we create a profile by entering the data of the training cohorts (*n* = 60). After that, prognosis is predicted by inputting the clinical parameters of test cohorts in the profile.

#### 2.4.3. Prediction Accuracy

Finally, we calculated the prediction accuracy rate by comparing the prediction result with the actual AIS at the discharge. We also compared the prediction accuracy of MLR analysis and ANNs.

## 3. Results

We inputted the clinical parameters in the profile. As basic information on patients, we adopted age at injury, sex, height, and body weight. Advanced age is associated with worse functional outcomes after SCI [21]. The presence or absence of OPLL that affects the outcome is added to the parameters [9,10].

To evaluate the extent of damage, we classified lesion types into 11 types: compression fracture, axis rotation dislocation fracture, atlas fracture, flexion teardrop fracture, dens fracture, anterior dislocation fracture, vertebral fracture, burst fracture, fracture of vertebral arch, and spinal cord injury without bone injury and others.

In addition to lesion types, we added MRI findings, such as sagittal low in high (+/−) and pre-vertebral hematoma (+/−), to the parameters. In the sagittal section of T2-enhanced MRI, sometimes, we can see a low signal area in hyperintensity. This imaging finding is known as a poor prognostic factor for patients with SCI [22].

We assessed neurological findings of SCI on admission according to International Standards for Neurological Classification of Spinal Cord Injury (ISNCSCI) and added its endpoints, such as AIS on admission, NLI (Neurological Level of Injury), ZPP (Zone of Partial Preservation), and ASIA total motor score, to the parameters.

**Figure 3:**
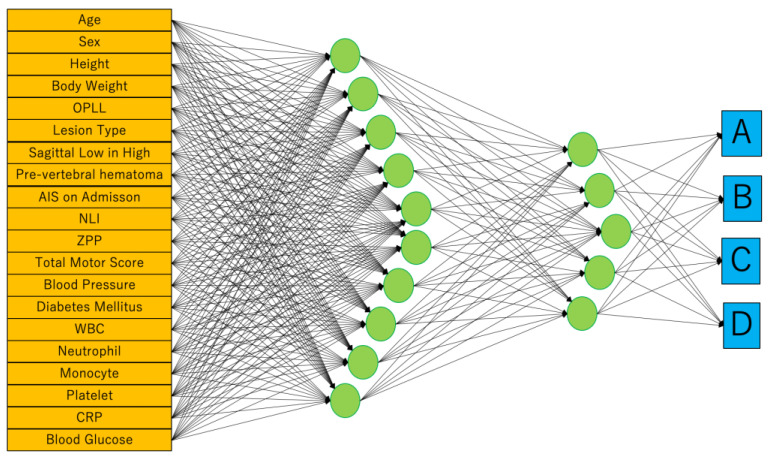
We adopted neural networks in JMP^®^ Pro version 14.2.0 (SAS Institute Inc., Cary, NC, USA). Weight Decay is used as a penalty method. It consists of input, 1st, 2nd, and output layers. The number of nodes in the 1st and 2nd layers are 5 and 10, respectively. The outputs are the ASIA Impairment Scale (AIS) at hospital discharge.

To reflect the patient’s general condition at the time of injury, blood pressure, presence of diabetes, and blood test results were included in the profile. In the results of the blood test, the white blood cell, neutrophil, monocyte, platelet, CRP, and blood glucose levels that were important for motor functional outcome after SCI were applied for parameters [7,23]. Finally, we built two predictive models using MLR and ANNs. Both models were created using these same 20 parameters (Figure 3).

Since parameters that are unprecedented in teacher data cannot be entered into the profile, there were four cases in which the prognosis could not be predicted in this study. Eventually, 16 out of 20 cases of test cohort were predictable.

**Figure 4:**
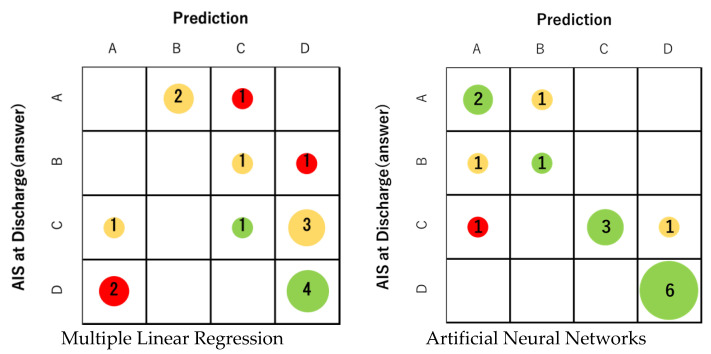
The prediction accuracy of MLR was 31.3% (5 correct answers). On the other hand, the correct answer rate of prediction using ANNs was 75.0% (12 correct answers). The correct answer rate of ANNs for each final AIS was 66% for AIS A, 50% for B, 60% for C, and 100% for D. The size of the circle represents the number of applicable cases. Green indicates that the prediction is correct, yellow indicates an error of one level, and red indicates an error of two or more levels.

First, profile prediction using MLR analysis was correct in only 5 out of 16 cases. The prediction accuracy was 31.3%, which was not sufficient (Figure 4). The correct answer rate for each final AIS grade was 0% for grades A and B, 20% for grade C, and 66% for grade D. On the other hand, the correct answer rate of prediction using ANNs was 75.0% (12 correct answers). The correct answer rate of ANNs was 66% for grade A, 50% for grade B, 60% for grade C, and 100% for grade D (Figure 4). In all grades, ANNs were able to obtain better prediction accuracy than MLR. For both MLR and ANNs, AIS grade C/D (ambulatory) at discharge tended to have a higher percentage of correct answers than AIS grade A / B (non-ambulatory). Regarding ANNs, the predictive accuracy of the ambulatory group (AIS grade C and D) was 81.8% in 9 out of 11 cases.

## 4. Discussion

In this study, we attempted to predict the prognosis of patients with cervical SCI from acute-phase clinical data using ANNs. This approach may allow for more accurate prognostic predictions, taking into account complex patient characteristics and multiple variables. Although it is generally believed that the prognosis of patients with cervical SCI becomes easier to predict as time passes, we focused on predicting the final prognosis from acute phase clinical data at the time of admission. In our study, we were able to predict prognosis using ANNs with an accuracy rate of 75%. The correct answer rate of MLR was 31.3%, which was significantly lower than the prediction accuracy of ANNs. Taking further consideration, if we consider ambulatory (AIS grades C and D)/non-ambulatory (grades A and B) as the primary endpoint rather than AIS, the correct answer rate was 62.5% in MLR, but the rate for ANNs was 93.4% (15 out of 16 cases answered correctly). Furthermore, looking at the correct answer rate for each grade of AIS, the higher the grade was, the higher the correct answer rate was for both MLR and ANNs. The main reason for this is thought to be that the final AIS grade tends to be ambulatory (grades C and D) because paralysis gradually improves with treatment. In this series of 16 cases, 11 cases were ultimately ambulatory, and only 5 cases were non-ambulatory.

In this series, the predictive accuracy of ambulatory was 93.4%. We believe it is extremely significant that we were able to predict whether more than 90% of patients would eventually be able to walk based on clinical data at the time of hospitalization. It is a well-known fact that whether or not a patient is able to walk greatly influences their life after being discharged from the hospital [24,25]. If walking ability can be predicted from clinical data in the acute phase, the prediction of outcomes can be used to inform rehabilitation goals and regimens, and it might lead to motivation when starting rehabilitation interventions [26]. In the early stages of cervical SCI, paralysis often occurs, and patients are extremely anxious [27]. Explaining the possibility of regaining walking ability will give patients great hope [28]. Furthermore, if the care team knows that there is a high possibility that the patient will eventually be able to walk, the ultimate goal of assistance and the extent of home renovations will naturally change [29].

Attempts to predict the prognosis of SCI patients using ANNs have been reported in the past. Belliveau T et al. predicted the prognosis after traumatic SCI using the Artificial Neural Network model [30]. They set the main outcome as self-reported ambulation and reported high predictive accuracy with a model that predicts ambulation status (>85% case classification accuracy). They also conducted a study to predict independence in self-care activities for people with SCI, with moderate accuracy (76–86% case classification accuracy). In their study, the cohort consisted of 3211 individuals who contributed data to the National Spinal Cord Injury Model Systems (SCIMS) Database between 2010 and 2014, but the input variables were very simple: age at the time of injury, sex, and ASIA exam manual motor testing scores. On the other hand, although the number of our training data is 60, we obtained relatively good results by increasing the input variables. In addition to basic data such as age and gender, 20 items included imaging findings, ASIA classification, blood sampling data during transportation, and the presence or absence of diabetes. Although the sample size was small, we were able to obtain relatively good prediction accuracy by increasing the number of clinical parameters. Additionally, due to the characteristics of the database we used, the primary endpoint was set to AIS grade, but this scoring alone can only provide a general understanding of the condition of patients with cervical SCI. However, by enriching the content of teacher data, it is also possible to predict factors that are directly linked to life after discharge, such as walking distance and self-care activities. Belliveau T et al. also compared ANNs and MLR analysis [30]. They reported that the performance of the model generated by ANN was equivalent to or exceeded that of the MLR model, and this result is consistent with our present results.

AI machine learning models, such as neural networks, often tend toward “overfitting” training data [31]. This is a concern because the model learns about noise and random fluctuations in the training data, which can reduce its generalizability to new data. To address this issue, we introduced weight regularization. Regularization is a technique that adds a penalty for large weights to the model’s loss function, which reduces overfitting to training data by encouraging the model to choose smaller weight values [32]. Specifically, this research adopted JMP’s Weight Decay regularization method. Weight Decay, also known as L2 regularization, appropriately constrains model parameters and suppresses overfitting by imposing penalties on large weight parameters. Appropriately avoiding overfitting is expected to improve the generalizability of the model and, as a result, improve prediction accuracy.

Many factors have been found to influence the prognosis of SCI [7,8,9,10,21,22,23]. This study significantly improved prognosis prediction ability by incorporating these factors into ANNs. In recent years, factors related to the prognosis of SCI have been discovered one after another [8,33]. ANNs can be modified by incorporating another clinical parameter. Although adding parameters to ANNs does not necessarily improve accuracy, ANNs have a lot of potential to be upregulated with new effective variables in the future [34].

Our future plans are to further increase the amount of training data and create predictive models that can handle more datasets. After that, we plan to publish the profile of the predictive model on the Internet so that many people can use it. We hope that this will help determine treatment strategies for cervical SCI.

Finally, research using AI is expected to increase at an accelerated pace. Various predictive tools using AI will likely be created in the field of SCI as well. For example, predictive models have the potential to become even more clinically useful tools by changing the predicted outcomes to various factors such as walking distance, degree of urinary assistance, and risk of dysphagia. By using various predictive models, patients can easily imagine their final standard of living.

## 5. Limitation

The logic used by ANNs to predict prognosis is a black box, and we cannot know the algorithm involved in prognosis, but it will not disturb the purpose of this study to predict the outcome of the patients of SCI.

We made predictions for the test set using a predictive model created based on training data. However, this model is also not predictive in all cases. Variables that are not included in the training data cannot be input into the predictive model. In other words, clinical parameters that are unprecedented in teacher data cannot be entered into the profile. In this study, 16 out of 20 cases were predictable. However, this problem can be solved by increasing the number of cases of training data.

The patients with cervical SCI in this study were hospitalized for a long period of time in a specialized facility at SIC, and during that period, they received highly specialized rehabilitation. Therefore, it is highly likely that the current predictive model will not be able to accurately predict the prognosis of patients in less specialized facilities or facilities with less time for rehabilitation. Ideally, it would be desirable for SCI patients across the country to be able to receive highly specialized rehabilitation, but this is extremely difficult to achieve. When predicting prognosis at other hospitals, it may be possible to solve this problem by incorporating rehabilitation time and quality as clinical parameters. In the future, we should consider ways to utilize this predictive model prospectively, but further research is required for this purpose.

## 6. Conclusions

ANNs developed from the data of the acute phase predicted the prognosis of the patients with cervical SCI more accurately than MLR analysis. Performing effective rehabilitation based on ANNs will lead to the improvement of the patient’s quality of life after discharge.

## Figures and Tables

**Table 1:** Participants and data splitting. * Height and body weight data were missing for three patients in the training group.

	All Patients (N = 80)	Training Group (N = 60)	Test Group (N = 20)
Age (range)	60.4 (18–83)	61.9 (18–83)	56.1 (18–79)
Sex			
Male (%)	64 (80.0)	47 (78.3)	17 (85.0)
Female (%)	16 (20.0)	13 (21.7)	3 (15.0)
Height (range)	165.8 (143–186) (N = 77 *)	164.9 (145–182) (N = 57 *)	168.3 (143–186)
Body weight (range)	64.4 (41–1147) (N = 77 *)	62.9 (41–86) (N = 57 *)	68.7 (41–114)
ASIA Impairment Scale on Admission			
A (%)	31 (38.8)	26 (43.3)	5 (25.0)
B (%)	15 (18.8)	10 (16.7)	5 (25.0)
C (%)	30 (37.5)	20 (33.3)	10 (50.0)
D (%)	4 (5.0)	4 (6.7)	0 (0.0)
NLI			
C1 (%)	2 (2.5)	2 (3.3)	0 (0.0)
C2 (%)	3 (3.8)	3 (5.0)	0 (0.0)
C3 (%)	11 (13.8)	8 (13.3)	3 (15.0)
C4 (%)	36 (45.0)	24 (40.0)	12 (60.0)
C5 (%)	17 (21.3)	14 (23.3)	3 (15.0)
C6 (%)	9 (11.3)	7 (11.7)	2 (10.0)
C7 (%)	0 (0.0)	0 (0.0)	0 (0.0)
C8 (%)	1 (1.3)	1 (1.7)	0 (0.0)
T1 (%)	1 (1.3)	1 (1.7)	0 (0.0)
ASIA total motor score on admission (range)	21.3 (0–72)	22.6 (0–72)	17.5 (1–44)
Lesion Type			
Compression fracture (%)	1 (1.3)	1 (1.7)	0 (0.0)
Axis rotation dislocation fracture (%)	1 (1.3)	1 (1.7)	0 (0.0)
Atlas fracture (%)	1 (1.3)	1 (1.7)	0 (0.0)
Flexion teardrop fracture (%)	3 (3.8)	2 (3.3)	1 (5.0)
Dens fracture (%)	2 (2.5)	2 (3.3)	0 (0.0)
Anterior dislocation fracture (%)	20 (25.0)	18 (33.3)	2 (10.0)
Vertebral fracture (%)	6 (7.5)	2 (3.3)	4 (20.0)
Burst fracture (%)	2 (2.5)	2 (3.3)	0 (0.0)
Fracture of the vertebral arch (%)	1 (1.3)	0 (0.0)	1 (5.0)
Spinal cord injury without bone injury (%)	42 (52.5)	30 (50.0)	12 (60.0)
Others (%)	1 (1.3)	1 (1.7)	0 (0.0)
OPLL (%)	23 (28.8)	20 (33.3)	3 (15.0)
Sagittal low in high (MRI) (%)	26 (32.5)	19 (31.7)	7 (35.0)
Pre-vertebral hematoma (MRI) (%)	53 (66.3)	38 (63.3)	15 (75.0)
Laboratory data			
WBC (×10³) (range)	10.8 (4.5–22.8)	10.9 (4.5–21.6)	10.5 (5.4–22.8)
Neut (×10³) (range)	9.1 (3.3–20.1)	9.2 (3.8–19.2)	8.7 (3.3–20.1)
Mono (×10²) (range)	4.93 (0.1–17.2)	4.8 (0.3–10.4)	5.4 (0.1–17.2)
Plate (×10⁴) (range)	18.8 (8.3–32.4)	18.1 (8.3–28.5)	20.9 (12.6–32.4)
CRP (range)	0.77 (0–11.4)	0.69 (0–5.0)	1.04 (0–11.4)
Blood glucose (range)	135 (76–376)	141 (76–376)	116 (84–171)
Diabetes mellitus (%)	22 (27.5)	19 (31.7)	3 (15)
Blood pressure			
Systolic (range)	119 (76–175)	117 (76–175)	122 (96–169)
Diastolic (range)	66 (25–97)	66 (25–97)	66 (43–91)
ASIA Impairment Scale at discharge			
A (%)	21 (26.3)	18 (30.0)	3 (15.0)
B (%)	8 (10.0)	6 (10.0)	2 (10.0)
C (%)	18 (22.5)	12 (20.0)	6 (30.0)
D (%)	33 (41.3)	24 (40.0)	9 (45.0)

## Data Availability

The data presented in this study are available on request from the corresponding author. The data are not publicly available due to restrictions on privacy and ethics.
